# Supplementary material for: Water quality assessment based on Nemerow pollution index method: A case study of Heilongtan reservoir in central Sichuan province, China
Source: PLoS One. 2022 Aug 18;17(8):e0273305. doi: 10.1371/journal.pone.0273305 (PMC9387827; doi:10.1371/journal.pone.0273305)
Supplement: Attachment — Submitted filename: renamed_e7765.docx [file pone.0273305.s001.docx]

**Dear Reviewer**s:

We appreciate your comments and ideas on our manuscript, which we used to make significant revisions to the original. In the amended version, all changes have been highlighted in the revised manuscript by formatting the text with yellow color. We hope the reviewers are pleased with our responses to the "comments" and the revisions we made to the original manuscript.

Reviewers' comments:

Reviewer's Responses to Questions 
 Comments to the Author

1. Is the manuscript technically sound, and do the data support the conclusions?

The manuscript must describe a technically sound piece of scientific research with data that supports the conclusions. Experiments must have been conducted rigorously, with appropriate controls, replication, and sample sizes. The conclusions must be drawn appropriately based on the data presented.  
 Reviewer #1: Yes 
 Reviewer#2: Yes 
 
 2. Has the statistical analysis been performed appropriately and rigorously?  
 
 Reviewer #1: Yes 
 Reviewer #2: Yes 
 
 3. Have the authors made all data underlying the findings in their manuscript fully available?

The PLOS Data policy requires authors to make all data underlying the findings described in their manuscript fully available without restriction, with rare exception (please refer to the Data Availability Statement in the manuscript PDF file). The data should be provided as part of the manuscript or its supporting information, or deposited to a public repository. For example, in addition to summary statistics, the data points behind means, medians and variance measures should be available. If there are restrictions on publicly sharing data—e.g.participant privacy or use of data from a third party—those must be specified. 
 
 Reviewer #1: Yes 
 Reviewer #2: Yes 
 
 4. Is the manuscript presented in an intelligible fashion and written in standard English?

PLOS ONE does not copyedit accepted manuscripts, so the language in submitted articles must be clear, correct, and unambiguous. Any typographical or grammatical errors should be corrected at revision, so please note any specific errors here. 
 
 Reviewer #1: Yes 
 Reviewer #2: No

**Responds:** Thanks for the very helpful comment. We have revised the language in the text to make it more fluent, standard and understandable
 
 5. Review Comments to the Author

Please use the space provided to explain your answers to the questions above. You may also include additional comments for the author, including concerns about dual publication, research ethics, or publication ethics. (Please upload your review as an attachment if it exceeds20,000 characters) 
 
 Reviewer #1: This paper uses the water quality monitoring data of Heilongtan Reservoir from 2019 to 2021, adopts the single factor pollution index method and Nemerow pollution index method to evaluate and analyze the water quality changes of Heilongtan Reservoir in three years, and explores the impact of human activities on the water quality of Heilongtan ReservoirIn addition, the article is easy to understand and has a clear framework system. However, there are some detailed mistakes in the article I have displayed as follows. I suggest that this paper can be better after some modifications.

Specific comments:
(1)The first sentence of the first paragraph of the Introduction is the same as the last sentence, please enrich the content.

**Responds:** Thanks to your valuable suggestion, we have removed the repetition in the first paragraph of the article, and have sorted out and revised the logic of the article here.

(2) The same phrase is used in multiple places in the text, please polish the text.

**Responds:** Thanks to your comment, we have checked and revised the article to remove repetitive language.

(3)In line 62, just use an example to prove the point is not enough.

**Responds:** Thank you for your suggestion and here we have added examples to support our point.

(4)Line 96,please reduce the use of short sentences and merge short sentences to express them more professionally.

**Responds:** Thanks for the very helpful comment. We have modified the phrase in line 96 to clarify the logic of the context and to make it more professional and readable.

(5)Line 108, about “Nemerrow pollution index method”, needs more introduction.
**Responds:** Thanks for the kind suggestion., the description of the method has been omitted here, and we have added the description of the Nemero Pollution Index method in the text to make it more complete

(6) The width of the column chart rectangle in the article is too wide, please adjust it to make it look more beautiful.

**Responds:** Thanks to your comments, We have modified the bar chart in the text and added data to the chart to make it look clearer and easier to understand.

(7)Please check the presentation of the article to avoid difficult to read questions.

**Responds:** Thank you for your comments, we have read the article carefully and corrected any incomprehensible or incomprehensible details in the text.

(8)Please check the use of passive voice in the article. There are some misuses of passive voice.

**Responds:** Thank you for pointing out the incorrect statements in the text. We have checked the text and corrected the incorrect use of the passive voice.

(9)Many expressions in the article are Chinese expressions, please modify them to make the expressions more native.

**Responds:** Thank you for your comments, the lack of language skills caused this problem and we have made changes to the Chinese English part of the text to bring it more in line with the requirements

(10)At the end of the paper, you can write the further meaning and some prospects of your research.

**Responds:** Thanks to your valid comments, we offered our thoughts on "whether the control criteria for the single factor index method could be increased to obtain a more rigorous assessment of water quality".

(11)The content of the article lacks innovation, please emphasize its innovation and research significance.

**Responds:** Thank you for your valuable comments. Due to the limitations of the evaluation method of the article, our innovative points were not reflected and we have modified some parts of the article in order to emphasize the significance of the article.

 Reviewer #2: Accurate evaluation of water quality is the basis of realizing river and lake health goals. In order toachieve accurate evaluation of river and lake water environment, Nemerow Pollution Index Method was used to evaluate water quality. The research method of this paper can be used as a reference for water quality evaluation of rivers and lakes. Where is the innovation of the system? What is the use value of the research method? Furthermore, there are more details (not limited) that should be discussed as following.

**Responds:** In the study of Heilongtan Reservoir, there are few studies using the Nemerow method to evaluate its water quality, and basically there is no analysis of its water quality through a large number of continuous testing data. Therefore, the evaluation of Heilongtan Reservoir through the data of several testing indicators for three years in this paper can grasp the changes of water quality in terms of specific pollution indicators, in order to provide a scientific basis for the utilization and protection of water quality.

1. In the abstract, add the research method and research conclusion.

**Responds:** Thanks to your comments, we have added a description of the single factor index method and the Nemerow pollution index method and the conclusions of the research in the abstract section.

2. We can’t get the conclusion of “Protection work is particularly important to the survival and development of human beings”.

**Responds:** Thank you very much for your suggestion, we have removed this illogical part and re-written this section after sorting out the logic.

3. Add the difference analysis among methods used for water environment quality evaluation.

**Responds:** Thank you for your suggested changes, we have added a comparison of differences in evaluation methods to the text

4. Include the limitation of the study in Introduction.

**Responds:** Thanks to your effective help, we have added to the introductory section about the limitation of the study

5. In the section of 2.1, add the current status of water quality in the Heilongtan Reservoir.

**Responds:** Thank you for raising the shortcomings of the article, we have added the current status of the reservoir water quality in the section of 2.1.

6. I recommend giving the data in the form of a table.

**Responds:** Thank you for your comment. This issue may be due to the fact that the graph in the text does not show you the exact values at a glance, so we have revised the graph to show the values on the graph to make it easier for the reader to understand the graph.

7. The paper lackscontent for discussion. The research process of the paper is detailed and the research results are fruitful, but we can not judge the correctness of the calculation results.

**Responds:** We would like to thank you for your comments. As the results of the evaluation method in the article are derived from the inherent formulae, the data required for the calculations can be found in the article and thus verified, and as regards the lack of some discussion, we remedy this by adding, for example, a comparison of methods and a reflection on this study.

8. Add the discussion on the rationality of research results and the applicability of research methods.

**Responds:** Thanks to your comments, we have added this discussion to the text.
 
 6. PLOS authors have the option to publish the peer review history of their article (what does this mean?). If published, this will include your full peer review and any attached files.


If you choose “no”, your identity will remain anonymous but your review may still be made public.


Do you want your identity to be public for this peer review? For information about this choice, including consent withdrawal, please see our Privacy Policy. 
 
 Reviewer #1: No 
 Reviewer #2: No 
 
 [NOTE: If reviewer comments were submitted as an attachment file, they will be attached to this email and accessible via the submission site. Please log into your account, locate the manuscript record, and check for the action link "View Attachments". If this link does not appear, there are no attachment files.]
