# Supplementary material for: Water quality assessment based on Nemerow pollution index method: A case study of Heilongtan reservoir in central Sichuan province, China
Source: PLoS One. 2022 Aug 18;17(8):e0273305. doi: 10.1371/journal.pone.0273305 (PMC9387827; doi:10.1371/journal.pone.0273305)
Supplement: Attachment — Submitted filename: Response to Reviewers.docx [file pone.0273305.s002.docx]

**Dear Editor and Reviewer**s:

We appreciate your comments and ideas on our manuscript, which we used to make significant revisions to the original. In the amended version, all changes have been highlighted in the revised manuscript by formatting the text with yellow color. We hope the reviewers are pleased with our responses to the "comments" and the revisions we made to the original manuscript.

Journal Requirements:
Please review your reference list to ensure that it is complete and correct. If you have cited papers that have been retracted, please include the rationale for doing so in the manuscript text, or remove these references and replace them with relevant current references. Any changes to the reference list should be mentioned in the rebuttal letter that accompanies your revised manuscript. If you need to cite a retracted article, indicate the article’s retracted status in the References list and also include a citation and full reference for the retraction notice.

**Responds:** Thanks for your kind reminder. We have checked the references.

Comments to the Author
1. If the authors have adequately addressed your comments raised in a previous round of review and you feel that this manuscript is now acceptable for publication, you may indicate that here to bypass the “Comments to the Author” section, enter your conflict of interest statement in the “Confidential to Editor” section, and submit your "Accept" recommendation.

Reviewer #1: All comments have been addressed

Reviewer #2: All comments have been addressed

2. Is the manuscript technically sound, and do the data support the conclusions?

The manuscript must describe a technically sound piece of scientific research with data that supports the conclusions. Experiments must have been conducted rigorously, with appropriate controls, replication, and sample sizes. The conclusions must be drawn appropriately based on the data presented.

Reviewer #1: Yes

Reviewer #2: Yes

3. Has the statistical analysis been performed appropriately and rigorously?

Reviewer #1: Yes

Reviewer #2: Yes

4. Have the authors made all data underlying the findings in their manuscript fully available?

The [PLOS Data policy](http://www.plosone.org/static/policies.action#sharing) requires authors to make all data underlying the findings described in their manuscript fully available without restriction, with rare exception (please refer to the Data Availability Statement in the manuscript PDF file). The data should be provided as part of the manuscript or its supporting information, or deposited to a public repository. For example, in addition to summary statistics, the data points behind means, medians and variance measures should be available. If there are restrictions on publicly sharing data—e.g. participant privacy or use of data from a third party—those must be specified.

Reviewer #1: Yes

Reviewer #2: Yes

5. Is the manuscript presented in an intelligible fashion and written in standard English?

PLOS ONE does not copyedit accepted manuscripts, so the language in submitted articles must be clear, correct, and unambiguous. Any typographical or grammatical errors should be corrected at revision, so please note any specific errors here.

Reviewer #1: Yes

Reviewer #2: Yes

6. Review Comments to the Author

Please use the space provided to explain your answers to the questions above. You may also include additional comments for the author, including concerns about dual publication, research ethics, or publication ethics. (Please upload your review as an attachment if it exceeds 20,000 characters)

Reviewer #1: I think the author has completed the most comments, However the structure of this paper is still need some work. I think there are too many tables in this paper. Can you try to draw more figures for this paper.

**Responds:** Thank you for your comments, we have revised the article with your guidance and added a figure to show the results of the single factor index method to make the article more readable.

Reviewer #2: Accurate evaluation of water quality is the basis of realizing river and lake health goals. In order to achieve accurate evaluation of river and lake water environment, Nemerow Pollution Index Method was used to evaluate water quality. The research method of this paper can be used as a reference for water quality evaluation of rivers and lakes.

**Responds:** Thank you for your approval of the article. With your suggestions, the structure of the article is more complete and the content is more in line with the requirements and standards.

7. PLOS authors have the option to publish the peer review history of their article ([what does this mean?](https://journals.plos.org/plosone/s/editorial-and-peer-review-process#loc-peer-review-history)). If published, this will include your full peer review and any attached files.

If you choose “no”, your identity will remain anonymous but your review may still be made public.

Do you want your identity to be public for this peer review? For information about this choice, including consent withdrawal, please see our [Privacy Policy](https://www.plos.org/privacy-policy).

Reviewer #1: No

Reviewer #2: No
